# Proteomic Analysis of FFPE Tissue Samples Identifies Potential Molecular Mechanisms Mediating Resistance to Radiotherapy in Rectal Cancer

**DOI:** 10.1021/acs.jproteome.5c00114

**Published:** 2025-06-27

**Authors:** Tobias Zott, Michael Wolf, Günter Plessl-Walder, Heinz Regele, Michael Bergmann, Samuel M. Meier-Menches, Christopher Gerner, Gerd R. Silberhumer, Andrea Bileck

**Affiliations:** † Department of General Surgery, 27271 Medical University of Vienna, Vienna 1090, Austria; ‡ Department of Analytical Chemistry, 224374 University of Vienna, Vienna 1090, Austria; § Vienna Doctoral School in Chemistry (DoSChem), University of Vienna, Waehringer Str. 42, Vienna 1090, Austria; ∥ Joint Metabolome Facility, University of Vienna and Medical University of Vienna, Vienna 1090, Austria; ⊥ Department of Pathology, 27271 Medical University of Vienna, Vienna 1090, Austria; # Institute of Inorganic Chemistry, University of Vienna, Vienna 1090, Austria

**Keywords:** autophagic activity, colorectal cancer, DNA damage repair, FFPE tissue, LC-MS, proteome profiling, radioresistance, radiotherapy, resistance mechanisms, tumor-associated stroma

## Abstract

Chemoradiation prior to surgery in locally advanced rectal cancer is the current standard therapy but is not effective in all rectal cancer patients. Prognostic markers supporting patient stratification with respect to clinical response would therefore be desirable. The aim of this study was to investigate pathophysiological mechanisms underlying radioresistance and to identify potential prognostic markers by comparative proteome profiling. Therefore, formalin fixed paraffin-embedded tissue (FFPE) samples from rectal tumors (*n* = 50) and normal control tissue (*n* = 39) of nonresponders and responders to neoadjuvant chemoradiation were analyzed. As a result, 1685 robustly identified proteins were further evaluated. Comparing tumor with corresponding control samples revealed 221 differentially expressed proteins (FDR < 0.05) with FTL, PCOLCE, and RCN3 being most striking in tumor tissue. CEACAM 1, 5, and 6, as well as MCM protein complex components, were significantly up-regulated in tumor tissue of nonresponders. The autophagic activity-related and DNA damage repair proteins TOM1, CAPNS1, TP53BP1, HS1BP3, as well as COTL1 and DCPS, discriminated non- and nearly complete from complete responders. In the tumor-surrounding tissue of nonresponders, the innate immune response-suppressing protein CD55 was found specifically up-regulated. These proteins may serve as prognostic markers and potential therapeutic targets, requiring further validation in prospective studies.

## Graphical abstract

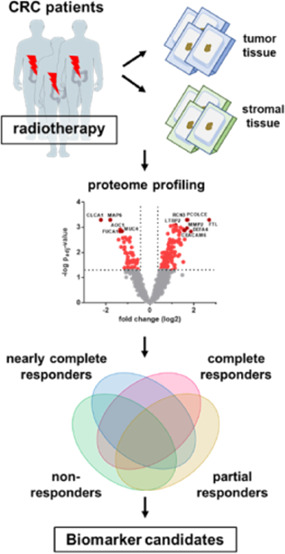

## Introduction

Colorectal cancer (CRC) is considered to be the third most common type of cancer in men and the second most common in women.^1^ One-third of these patients suffer from rectal cancer.^2^ Locally advanced tumors are usually treated with neoadjuvant radiotherapy and/or chemotherapy to reduce the risk of locoregional recurrence and to achieve higher rates of sphincter preservation.^2^ Clinical complete response (cCR) can be seen in 10–40% of rectal cancer patients; however, only half of these correlate with pathological complete responses (pCR).^3,4^ Clinical complete response might even prevent surgical resection of the rectum if the “watch and wait strategy” is adopted.^5^ Nevertheless, most patients show only partial or no response to neoadjuvant treatment. Further, radiotherapy is associated with significant side effects, including sexual dysfunction, loss of sphincter function, gastrointestinal complications, excessive scarring, and urinary tract problems.^6^

Total neoadjuvant therapy (TNT) and immunotherapy have gained interest in recent years as novel treatment strategies. TNT has resulted in higher pathologic complete response rates and improved outcomes compared to standard neoadjuvant chemoradiotherapy.^7,8^ Furthermore, immune checkpoint inhibitors show promising results in combination with neoadjuvant chemoradiotherapy (nCRT).^9^

The pathophysiological mechanisms responsible for resistance to radiotherapy are still not fully understood, and current research is typically based on *in vitro* model systems. The main reported molecular mechanisms leading to radiation resistance are DNA damage repair and inhibition of cell death mechanisms as well as protective effects of the tumor microenvironment.^10^ Especially, the contribution of the stroma and the interplay between tumor and stroma during radiation therapy are of great importance to better understand the mechanisms of radiation resistance.^11^ Furthermore, major efforts were undertaken to identify markers predicting response to nCRT^12–14^ as well as to identify predictive biomarkers for radioresistant rectal cancer,^15^ but to the best of our knowledge, no reliable clinical strategy to predict therapeutic success to chemoradiation in colorectal cancer patients has been established yet.

Comprehensive proteomics has already been proven as a powerful tool to identify marker signatures and predict therapeutic responses in melanoma^16,17^ as well as to allow detailed analysis of the tumor microenvironment.^18,19^ Previously, tissue proteomics allowed us to assess the functional state of cancer-associated fibroblasts in the tumor-surrounding tissue using fresh frozen needle biopsies of breast cancer patients.^20^ In recent years, formalin-fixed paraffin-embedded (FFPE) tissue has gained traction for the discovery of biomarkers by LC-MS-based proteome profiling due to technical advances in sample preparation as well as LC-MS performance regarding sensitivity and robustness.^21^ FFPE tissue proteomics has already been successfully applied to investigate various cancer types.^22^ Using this technology, key proteins associated with CRC and the risk of CRC metastasis to lymph nodes,^23,24^ as well as stratification markers for small cell lung cancer (SCLC),^25^ were identified. In ovarian clear cell carcinoma, a promising potential biomarker for chemotherapy success was proposed based on the analysis of FFPE tissue.^26^ Recently, the response to nCRT in locally advanced rectal cancer was investigated using data-independent acquisition mass spectrometry (DIA-MS) leading to the identification of several potential predictive marker molecules.^27^ Further, Tüshaus et al. published the first pan-cancer FFPE proteomics study, consisting of 1220 tumor samples derived from six cancer entities, as a web resource.^21^ Due to technological advances, the analysis of laser capture microdissected FFPE tissue by means of LC-MS has also become possible, enabling spatially resolved proteomics.^28^

The possibility of long-term storage of FFPE tissue samples of various patient cohorts in biobanks fostered the use of this sample type for retrospective biomarker discovery studies by LC-MS-based proteome profiling. Indisputably, this has already been proven successful. The present study aims to forge ahead the potential of FFPE regarding its information content, focusing on the investigation of the pathophysiological mechanisms underlying radioresistance in CRC. A better understanding of these cellular mechanisms, not only within the tumor tissue but also in the tumor-surrounding tissue, may support the identification of prognostic molecules as well as potential therapeutic targets. Therefore, not only tumor tissue samples of responders and nonresponders to radiation therapy were analyzed but also tumor-surrounding (normal control) tissue. This experimental design allowed us to investigate significant proteome differences between responders and nonresponders, highlighting potential resistance mechanisms during chemoradiation including tumor as well as tumor-surrounding tissue.

## Experimental Section

### Study Design

Fifty patients diagnosed with rectal cancer who underwent surgery at the Medical University of Vienna, Department of General Surgery, between 2010 and 2017 were included in this study. The study was approved by the Ethics Committee of the Medical University of Vienna (EC No.: 1330/2020) and conducted in accordance with both the Declarations of Helsinki and Istanbul. All participants gave their informed consent.

The tumor specimens were postoperatively processed according to the local protocol of the Department of Pathology. At the time of our analysis, the tissue samples of the study patients were collected from the pathological archive and prepared for our proteomics analysis as described below. The control tissue samples were collected from the same surgical specimens and confirmed as normal tissue by the pathologist. Patient data were obtained from a local patient documentation system.

Response to neoadjuvant therapy (NAT) was defined according to the American Joint Committee on Cancer and College of American Pathologists regression grade (AJCC/CAP). AJCC/CAP grade 0 (complete response), grade 1 (nearly complete response, <10% vital tumor cells), and grade 2 (partial response, 10–50% vital tumor cells) were defined as “responders”, whereas patients with AJCC/CAP grade 3 (poor or no response, >50% vital tumor cells) were defined as “nonresponders”.^29^ Patients marked as “responders” were compared to “nonresponders”. Furthermore, subgroup analyses of the different AJCC/CAP grades were performed.

First, tumor tissue was compared to healthy tissue to identify potential differences in the proteome and validate the tissue specimens by determining known oncological proteins. Healthy tissue samples were harvested from the same surgical specimens and confirmed as normal tissue sections by the pathologist, thus leading to their use as control tissue samples for proteome analyses. Second, differences in the proteome between responders and nonresponders to radiation therapy were investigated. Lastly, the impact of the tumor-surrounding control tissue (stroma) on mechanisms underlying radioresistance was examined. An overview of the used samples can be seen in Figure 1. Out of a total of 50 tumor patients, paired tissue (control and tumor) samples were available from 39 patients. Therefore, these samples were used for the paired statistical analysis of tumor and control FFPE samples. Regarding the analysis of different response groups to radiation, all 50 tumor samples were included in the statistical analysis. Regarding complete responders, in this study, the term “tumor tissue” was used for simplicity, but tumor tissue from these patients mainly consisted of scar tissue.

**Figure 1:**
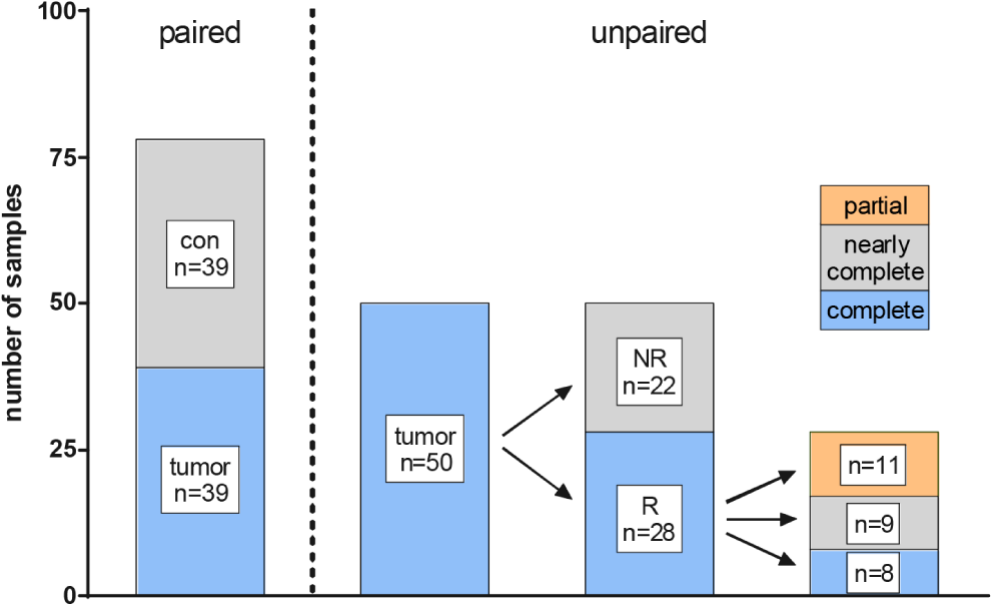
Overview of the samples used for statistical analysis. R, responders; NR, nonresponders.

### Sample Preparation

Formalin-fixed paraffin-embedded (FFPE) rectal tumor and control tissue samples were transferred into 15 mL Falcon tubes and incubated with 250 μL of lysis buffer (8 M urea, 50 mM TEAB, 5% SDS) overnight at 50 °C. Thereafter, the samples were sonicated using an ultrasonic stick and centrifuged at 2500 *g* for 5 min. Then, the samples were placed in a 50 °C-heated ultrasonic bath by applying 10% power for 1 h before they were vortexed and centrifuged at 2000 *g* for 1 min. This step was performed three times in total. Finally, the samples were sonicated again using an ultrasonic stick and centrifuged at 2000 *g* for 5 min before they were transferred into Eppendorf tubes. The samples were then incubated at 80 °C and 1000 rpm for 1 h on a thermomixer. Afterward, the samples were centrifuged at 10,000 *g* for 5 min. The clear soluble fraction was transferred into a new Eppendorf tube and centrifuged again at 15,000 *g* for 5 min before the protein concentration of all samples was determined using the BCA assay.

Enzymatic protein digestion was achieved by applying a protocol using the S-trap technology with minor adaptions.^30^ Briefly, 20 μg of protein was reduced and alkylated by the addition of 64 mM DTT and 486 mM IAA, respectively. After the addition of trapping buffer (90% v/v methanol, 0.1 M triethylammonium bicarbonate), the samples were loaded onto S-trap cartridges and thoroughly washed (3× MeOH/chloroform, 50:50 (v/v), and 3× trapping buffer). Subsequently, the samples were digested using Trypsin/Lys-C Mix at 37 °C for 2 h. Thereafter, peptides were eluted, dried, and stored at −20 °C until LC-MS analyses.

### LC-MS/MS Analyses

LC-MS/MS analyses were performed as previously published.^16,31,32^ Briefly, dried peptide samples were reconstituted in 5 μL of 30% formic acid (FA) containing 4 synthetic standard peptides and diluted with 40 μL of loading solvent (97.95% H_2_O, 2% ACN, 0.05% trifluoroacetic acid). Of this solution, 10 μL were injected into the Dionex Ultimate 3000 nano high-performance liquid chromatography (HPLC) system (Thermo Fisher Scientific). Peptides were concentrated on a precolumn (2 cm × 75 μm C18 PepMap100; Thermo Fisher Scientific) at a flow rate of 10 μL/min, using mobile phase A (99.9% H_2_O, 0.1% FA). Afterward, peptides were separated by elution from the precolumn to an analytical column (25 cm × 75 μm Aurora Series emitter column (Ionopticks)) applying a flow rate of 300 nL/min and using a gradient of 8–40% mobile phase B (79.9% ACN, 20% H2O, 0.1% FA) over 95 min, resulting in a total LC run time of 135 min including washing and equilibration steps. Mass spectrometric analyses were performed using a timsTOF Pro mass spectrometer (Bruker) equipped with a captive spray ion source run at 1650 V and operated in the Parallel Accumulation—Serial Fragmentation (PASEF) mode.

### LC-MS/MS Data Analysis

The publicly available software package MaxQuant 1.6.17.0, running the Andromeda search engine, was used for protein identification as well as label-free quantification (LFQ).^33^ Therefore, TDF data generated from the Bruker timsTOF Pro mass spectrometer were searched against the human SwissProt database (version 2019_12_14 with 20380 entries). Search parameters included an allowed peptide tolerance of 25 ppm, a maximum of 2 missed cleavages, carbamidomethylation on cysteines as a fixed modification, as well as methionine oxidation and N-terminal protein acetylation as variable modifications. Furthermore, a minimum of two peptide identifications per protein, with at least one of them unique, was used as a search criterion. In addition, the “match between runs” option was applied using a 0.7 min match time window and a match ion mobility window of 0.05 as well as a 20 min alignment time window and an alignment ion mobility of 1. An FDR of ≤0.01 was set for all peptide and protein identifications.

Data evaluation as well as statistical analysis was performed using Perseus (version 1.6.14.0).^34^ Therefore, proteins were filtered for reversed sequences as well as common contaminants, and label-free quantification (LFQ) intensity values were transformed (log_2_(*x*)). In the first step, samples from 39 patients were annotated according to the following groups: control tissue and tumor tissue (*n* = 78). This sample matrix was used for paired *t* tests statistics to compare tumor versus control tissue. In a second data matrix, all analyzed tumor samples (*n* = 50) were annotated according to the following groups: nonresponder (AJCC/CAP grade 3) and responder (AJCC/CAP grades 0, 1, and 2) as well as nonresponder (AJCC/CAP grade 3), partial responder (AJCC/CAP grade 2), nearly complete responder (AJCC/CAP grade 1), and complete responder (AJCC/CAP grade 0) to allow comparison within the subgroups of responders. This sample matrix was used for unpaired *t* tests statistics to compare nonresponders versus responders or nonresponders versus the subgroups of responders. Prior to statistical analysis, proteins in both data matrices were filtered for a minimum of positive identifications (50%) in at least one group, and imputation of missing values by normal distribution was performed. Two-sided *t* tests between respective groups were performed applying an FDR of 0.05 and an S0 of 0.1, whereby S0 controls the relative importance of the *t* test *p-*value and the difference between the means. In addition, GraphPad Prism Version 6.07 (2015) was used to generate volcano plots and histograms.

The statistical tests used for the demographic variables were nonparametric tests for independent samples and Pearson’s chi-squared test.

## Results

The study population consisted of 50 patients diagnosed with rectal cancer who underwent surgery at the Medical University of Vienna, Department of General Surgery, between 2010 and 2017. Characteristics of the study cohort can be found in Table 1. Prior to surgery, all patients received neoadjuvant radiotherapy with or without chemotherapy. Thirteen patients (26%) underwent neoadjuvant radiotherapy alone, whereas 37 patients (74%) also received chemotherapy with either Xeloda (*n* = 31, 83.8%), Xelox/Avastin (*n* = 4, 10.8%), or Folfoxiri (*n* = 1, 2.7%, 1 missing). Out of the 28 (56%) responders, 8 (16%) patients showed a complete pathological response (AJCC/CAP grade 0), 9 (18%) a nearly complete response (AJCC/CAP grade 1), and 11 (22%) a partial pathological response (AJCC/CAP grade 2). Most patients underwent open surgery (80%) with an extirpation rate of 32%.

**Table 1:** Demographics of Study Population^a^

Parameter	Total (*n* = 50)	Responder (*n* = 28, 56%)	Nonresponder (*n* = 22, 44%)	*p*-Value
Sex				0.108
Male	37 (74%)	18 (64.3%)	19 (86.4%)	
Female	13 (26%)	10 (35.7%)	3 (13.6%)	
Age (Years, Q1–Q3)	68.2 (59.8–72.8)	65.9 (56.0–73.0)	70.5 (62.7–72.7)	0.458
BMI (kg/m^2^, Q1–Q3)	24.5 (22.0–29.0)	25.5 (22.5–28.7)	23.1 (20.6–29.1)	0.149
Distance to anal verge (cm, Q1–Q3)	6 (3–8)	5 (4–8)	7 (3–8)	0.443
UICC				0.194
I	8 (16%)	6 (21.4%)	2 (9.1%)	
II	20 (40%)	8 (28.6%)	12 (54.5%)	
III	12 (24%)	6 (21.4%)	6 (27.3%)	
IV	2 (4%)	0 (0%)	2 (9.1%)	
Complete response	8 (16%)	8 (28.6%)	0 (0%)	
NAT				0.009*
RT	13 (26%)	3 (10.7%)	10 (45.5%)	
CRT	37 (74%)	25 (89.3%)	12 (54.5%)	
Radiation protocol				0.012*
Short course	15 (30%)	4 (14.3%)	11 (50.0%)	
Long course	35 (70%)	24 (85.7%)	11 (50.0%)	
Type of surgery				
Laparoscopic	10 (20%)	5 (17.9%)	5 (22.7%)	0.732
Open	40 (80%)	23 (82.1%)	17 (77.3%)	
Extirpation (yes)	16 (32%)	6 (21.4%)	10 (45.5%)	0.071
Survival				
OS (d, Q1–Q3)	958 (395–2089)	1312 (713–2020)	684 (328–2316)	0.427
Death (yes)	28 (56%)	13 (46.4%)	15 (68.2%)	0.124
Adjuvant therapy				0.121
Yes	21 (42%)	9 (32.1%)	12 (54.5%)	
No	21 (42%)	14 (50%)	7 (31.8%)	
Data not available	8 (16%)	5 (17.9%)	3 (13.6%)	

a BMI, body mass index; UICC, Union Internationale Contre le Cancer; NAT, neoadjuvant therapy; RT, radiotherapy; CRT, chemoradiotherapy; OS, overall survival; *p value <0.05. Responders were defined as grade 0–2 according to the AJCC/CAP grading.

For a comparison of tumor and control FFPE tissue, proteomic data from 39 patients were submitted to paired statistical analysis (Figure 1). A total number of 1354 proteins were robustly identified, whereof 221 proteins showed significantly different abundance values when comparing rectal tumor and control samples (Table S1). Thereof, 174 proteins were significantly higher in tumor tissue compared to 47 proteins enriched in control tissue samples. Significantly up-regulated proteins in tumor tissue included ferritin light chain (FTL, adj. *p*-value ≤ 0.0005), procollagen C-endopeptidase enhancer (PCOLCE, adj. *p*-value ≤ 0.0005), and reticulocalbin 3 (RCN3, adj. *p*-value ≤ 0.0005). Significantly higher expressions of calcium-activated chloride channel regulator 1 (CLCA1, adj. *p*-value ≤ 0.0005) and microtubule-associated protein 6 (MAP6, adj. *p*-value ≤ 0.0005) were detected in control tissue samples (Figure 2A).

**Figure 2:**
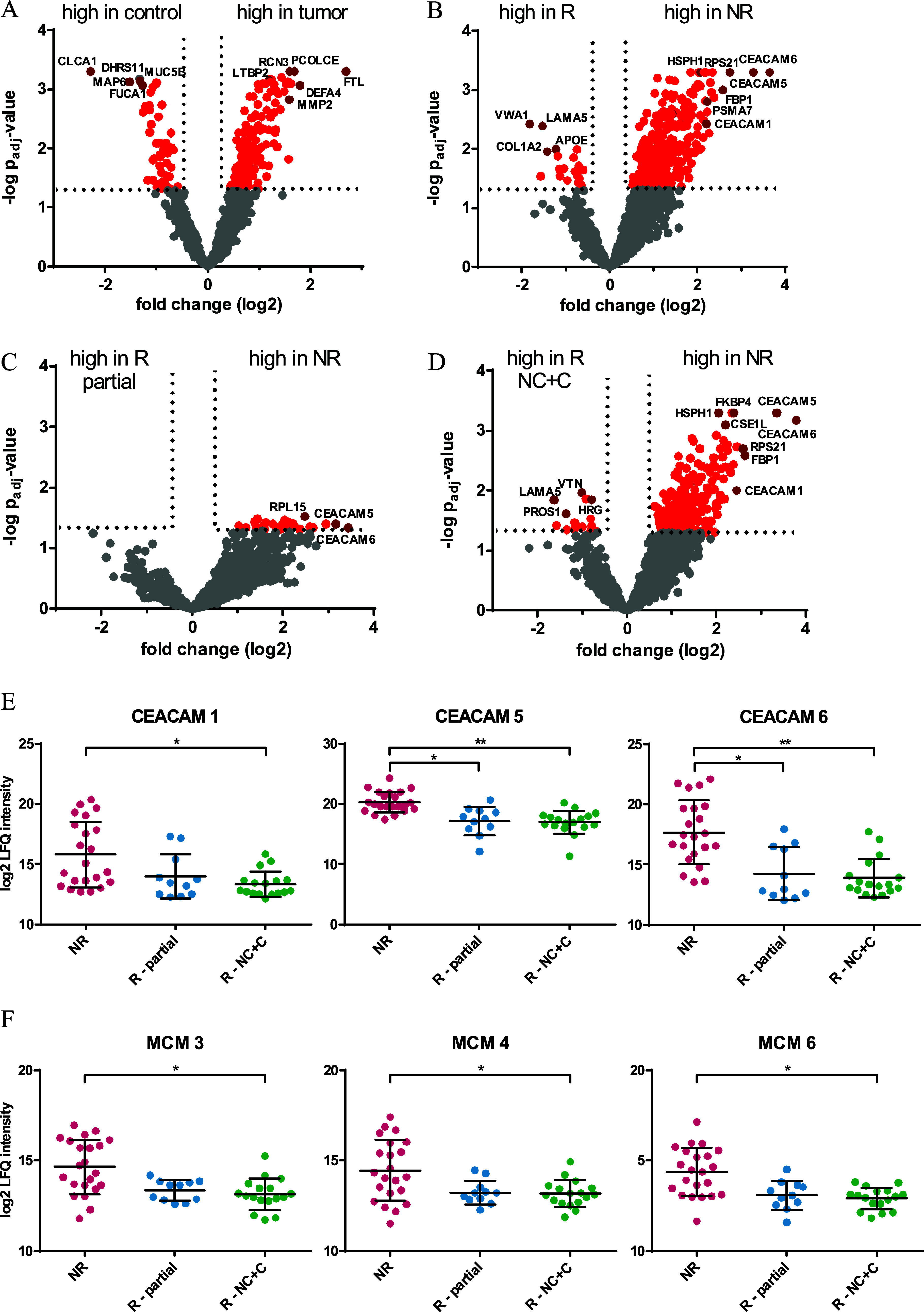
Comparison of protein abundances in colorectal tissue samples. Volcano plots illustrating significantly regulated proteins in (A) tumor versus control tissue, (B) tumor tissue from nonresponders (NR) versus responders (R) to radiation therapy as well as (C) nonresponders versus partial responders and (D) nonresponders versus nearly complete and complete responders (NC + C). (E,F) Dot plots of specific proteins showing log2 label-free quantification (LFQ) values for tumor tissue samples of each patient group, i.e., NR, nonresponders; R – partial, partial responders, and R – NC + C, nearly complete and complete responders. *Adj. *p*-value ≤ 0.05, **adj. *p*-value ≤ 0.005.

Subsequently, a data analysis of tumor tissue samples derived from all 50 patients was performed. Therefore, tumor samples were annotated according to the patients response to neoadjuvant therapy. Out of 50 patients suffering from rectal tumor, 28 showed a response (R) and 22 showed no response (NR) to neoadjuvant therapy. Responders were further divided into complete responders (*n* = 8), nearly complete responders (*n* = 9), and partial responders (*n* = 11; Figure 1). Tumor samples from complete responders mainly consisted of scar tissue. In this data set, a total number of 1685 proteins were identified and submitted to a statistical comparison of radiation response types (Table S2). Regarding the comparison of nonresponders versus responders, the abundance values of 381 proteins were significantly different (Figure 2B). Thereof, the abundance of 362 proteins was significantly higher in nonresponders and 19 in responders, as demonstrated in Figure 2. Carcinoembryonic antigen-related cell adhesion molecules 5 and 6 (CEACAM, adj. *p*-value ≤ 0.0005 each) as well as heat shock protein 105 kDa (HSPH1, adj. *p*-value ≤ 0.0005) showed the highest significance in nonresponders. Gene Ontology term enrichment analysis of significantly up-regulated proteins in nonresponders revealed cytoplasmic translation as the main enriched biological processes with an adj. *p*-value of 1.2 × 10^–19^. von Willebrand factor A domain-containing protein 1 (VWA1, adj. *p*-value = 0.0038), laminin subunit alpha-5 (LAMA5, adj. *p*-value = 0.0041), and collagen alpha-2­(I) chain (COL1A2, adj. *p*-value = 0.0111) belonged to the most prominently significantly up-regulated proteins in responders.

**Figure 3:**
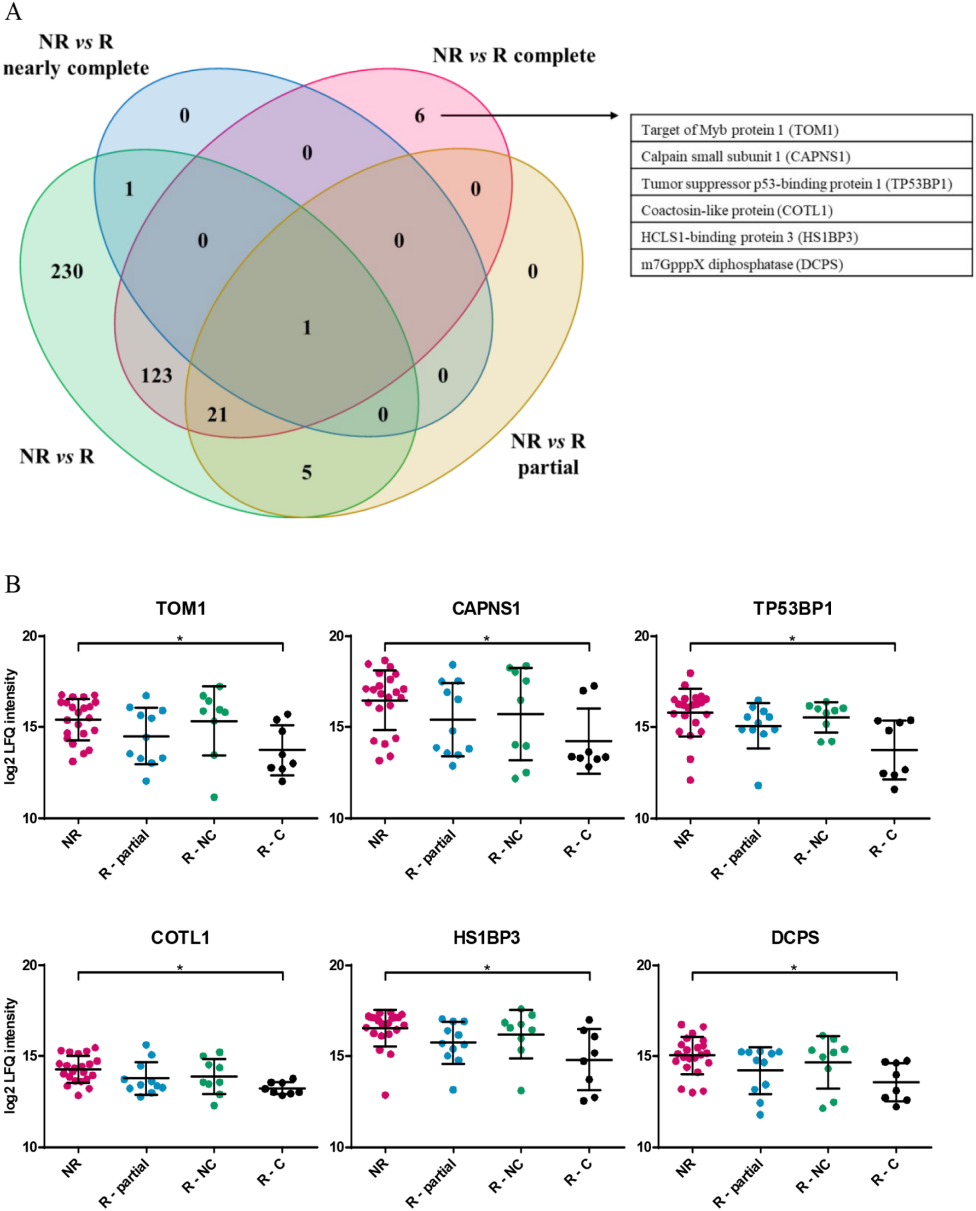
Specific protein signature in complete responders. (A) Venn diagram of all significantly regulated proteins between nonresponders and responders as well as nonresponders and the different response types. (B) Dot plots of specific proteins showing log2 label-free quantification (LFQ) values for tumor tissue samples of each patient group, i.e., NR, nonresponders; R – partial, partial responders; R – NC, nearly complete responders; and R-C, complete responders. *Adj. *p*-value ≤ 0.05.

When comparing nonresponders (*n* = 22) and a subgroup of responders, i.e., partial responders (*n* = 11, Figure 2C), 27 proteins were significantly higher abundant in nonresponders, for example, 60S ribosomal protein L15 (RPL15, adj. *p*-value = 0.03), CEACAM6 (adj. *p*-value = 0.046), and CEACAM5 (adj. *p*-value = 0.04). Comparison of tissue samples from nonresponders (*n* = 22) versus complete and nearly complete responders, grouped to represent “good” responders (*n* = 17), resulted in 264 significantly regulated proteins with 249 proteins higher abundant in nonresponders and 15 proteins in complete responders (Figure 2D). Significantly up-regulated proteins in nonresponders were CEACAM5 (adj. *p*-value ≤ 0.0005), HSPH1 (adj. *p*-value ≤ 0.0005), and 40S ribosomal protein S7 (RPS7, adj. *p*-value ≤ 0.0064), while histidine-rich glycoprotein (HRG, adj. *p*-value ≤ 0.0141), laminin subunit alpha-5 (LAMA5, adj. *p*-value ≤ 0.0144), and vitamin K-dependent protein S (PROS1, adj. *p*-value ≤ 0.0243) were the most up-regulated proteins in the “good” responder group. The statistical comparison of tumor tissue derived from nearly complete and complete responders to radiation therapy did not reveal any significant changes in protein abundance.

Remarkably, the group of cancer-related proteins, i.e., CEACAM1, CEACAM5, and CEACAM6 as well as the minichromosome maintenance complex components MCM3, MCM4, and MCM6 showed clear protein abundance patterns related to the radiation response (Figure 2E,F). While protein abundances were highest in nonresponders, abundance levels were significantly lower in partial responders, as well as in nearly complete and complete responders to radiation therapy.

To identify proteins specifically regulated in one of the subgroups regarding the response type, a Venn diagram with all significantly regulated proteins was created (Figure 3A). The abundances of only 6 proteins were significantly regulated when comparing nonresponders with complete responders but not when comparing nonresponders with nearly complete or partial responders. These proteins encompass the target of Myb protein 1 (TOM1, adj. *p*-value = 0.0429), calpain small subunit 1 (CAPNS1, adj. *p*-value = 0.0420), tumor suppressor p53-binding protein 1 (TP53BP1, adj. *p*-value = 0.033), coactosin-like protein (COTL1, adj. *p*-value = 0.0416), HCLS1-binding protein 3 (HS1BP3, adj. *p*-value = 0.0387), and m7GpppX diphosphatase (DCPS, adj. *p*-value = 0.0453), as demonstrated in Figure 3B.

Furthermore, we investigated differences in the tumor-surrounding tissue (control) between nonresponders and responders to radiation therapy. Therefore, paired statistical analysis of tumor and control tissue of patients was performed separately for nonresponders and responders (Figure 4). Remarkably, while the abundance of 152 proteins was significantly different between tumor and control tissue samples of nonresponders (Figure 4A), only 74 proteins were significantly different in tumor and control tissue samples of responders to radiation therapy (Figure 4B).

**Figure 4:**
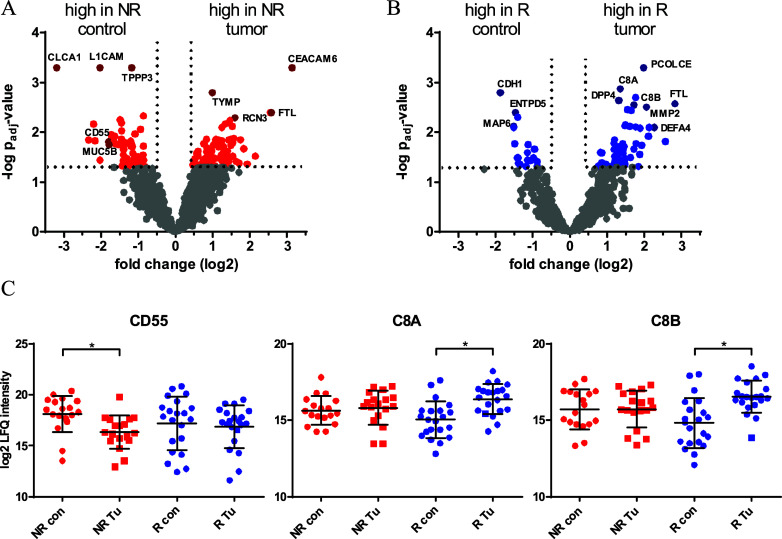
Contribution of the tumor-surrounding tissue. Volcano plots illustrating significantly regulated proteins between tumor and control tissue samples of (A) nonresponders (NR, *n* = 18) and (B) responders (R, *n* = 21) to radiation therapy. (C,D) Dot plots of specific proteins showing log2 label-free quantification (LFQ) values for control (con) and tumor (Tu) tissue of nonresponders (NR) and responders (R) to radiation. *Adj. *p*-value ≤ 0.05.

The complement decay-accelerating factor (CD55), known to be highly expressed in the tumor environment and to play a role in the suppression of adaptive immune responses,^35,36^ was significantly up-regulated in the control tissue of nonresponders (Figure 4C). The up-regulation of complement-associated proteins like complement component C8 alpha and beta chain (C8A and C8B) in the tumor tissue of responders but not in the tumor tissue of nonresponders might result from the higher levels of CD55 in the stroma of nonresponders, thereby inhibiting the central driver of the complement cascade, C3 convertase.^37^

## Discussion

It is still an issue of debate whether nonresponders benefit from neoadjuvant radiation in rectal cancer at all or if neoadjuvant radiation should be excluded from therapeutic strategies in these patients. Therefore, molecular predictors for therapy responses are of great interest. Comprehensive proteome profiling has already been proven as a powerful tool to identify a marker signature as well as to predict therapeutic response in melanoma.^16,17^ Moreover, LC-MS-based proteomics allows a detailed analysis of the tumor microenvironment to better understand the pathomechanisms taking place during tumor progression.^18,19^

In this study, we evaluated the suitability of surgical FFPE tissue samples of rectal cancer patients receiving neoadjuvant radiation therapy for LC-MS-based proteome profiling in order to investigate differences in protein abundance between responders and nonresponders to radiation therapy. To test the power of this strategy, we first analyzed tumor and healthy tissue samples of rectal cancer patients with regard to known tumor markers. As a second step, we analyzed surgical tumor tissue samples of responders and nonresponders after radiation therapy in order to characterize potential marker proteins predicting response to neoadjuvant radiation therapy. The availability of tumor-surrounding control tissue further supported the investigation of the stromal impact on radioresistance mechanisms.

We were able to identify a wide variety of known oncological proteins in the tumor tissue samples, successfully demonstrating the suitability of FFPE samples for the LC-MS-based proteome profiling approach (Figure 2A). As an example, FTL, RCN3, and PCOLCE belong to the most up-regulated proteins in tumor tissue samples. While RCN3 has already been described to play an important role in human colorectal cancer,^38^ FTL and PCOLCE were associated with poor prognosis in various cancer types.^39–42^ Interestingly, latent-transforming growth factor beta-binding protein 2 (LTBP2), which was found to be significantly up-regulated in tumor tissue samples, has already been identified in cancer-associated fibroblasts by proteome profiling and serves as a prognostic factor in colorectal cancer.^43^ Chloride channel accessory 1 (CLCA1) was found to be significantly enriched in control tissue samples of colorectal cancer patients. Chen et al. described that high expression of CLCA1 is generally linked to poor therapeutic response and survival outcomes in rectal cancer patients who received NAT prior to surgery.^44^

The comparison of proteome profiles of nonresponders and responders to chemoradiation demonstrated a large number of up-regulated proteins in nonresponders compared to different subtypes of responders (Figure 2B–D). This may be a consequence of the cell composition of tumor tissue from complete responders consisting mainly of scar tissue. Nevertheless, proteins of the CEACAM family were strongly up-regulated in nonresponders, giving rise to their potential role in radioresistance (Figure 2E). CEACAM 1, 5, and 6 were previously described as important cell adhesion molecules and as promising biomarkers in melanoma, lung, colorectal, and pancreatic cancers.^45–47^ Furthermore, the minichromosome maintenance complex components MCM3, MCM4, and MCM6 showed clear protein abundance patterns based on the radiation response (Figure 2F) and have already been described in colorectal cancer progression.^48–50^

In order to identify specific proteins distinguishing nonresponders from complete responders and potentially serving as prognostic molecules in future, a comparison of all significantly regulated proteins between different response types to chemoradiation was performed. This resulted in the identification of six proteins characteristic for nonresponders, namely TOM1, CAPNS1, TP53BP1, COTL1, HS1BP3, and DCPS (Figure 3B). Thus, high expression levels of these molecules may serve as an indicator for insensitivity to chemoradiation. Potentially due to a different classification system of the patient cohort, our results have little overlap with a previously published study identifying proteins encoded by the genes SMPDL3A, PCTP, LGMN, SYNJ2, NHLRC3, GLB1, and RAB43 as potential predictive markers for unfavorable treatment outcomes.^27^

TOM1 functions as an adapter protein for intracellular membrane trafficking and endosomal cargo sorting. It has already been described to play an important role in autophagy, immune response, and neuroinflammation. Further, alterations of TOM1 function have been associated with cancer progression.^51^ Additionally, it has been shown that by using MiR-126, a microRNA targeting TOM1, colon cancer progression could be successfully inhibited and that MiR-126 levels are strongly related to patient prognosis.^52^ Next to TOM1, CAPNS1 was specifically up-regulated in nonresponders compared to complete responders. In general, the calpain system is required for a plethora of physiological and pathophysiological processes like cell cycle, proliferation, migration, autophagy, apoptosis, and tumorigenesis. It catalyzes and regulates proteolysis of specific substrates, and it has been described to mediate cancer progression. Thus, inhibition of calpain activity may be a potential therapeutic strategy to target cancer cell survival, invasion, and chemotherapy resistance.^53^ An integrative analysis of autophagy-related genes identified CAPNS1 as a potential novel prognostic biomarker of melanoma which promotes the malignancy via Notch signaling pathway.^54^ Furthermore, Ye et al. described CAPNS1 as a key prognostic autophagy-related gene for hepatocellular carcinoma.^55^ In colorectal cancer, CAPNS1 plays a tumorigenic role by increasing MAPK7 expression, which is associated with the promotion of cancer cell proliferation.^56^ Another autophagy-related protein, namely HS1BP3, was found to be characteristic for nonresponders. It has been already shown that HS1BP3 inhibits autophagy by regulation of phospholipase D.^57^ Further, in hepatocellular carcinoma, HS1BP3 was described as a tumor-promoting factor, and high expression of HS1BP3 is significantly associated with poor prognosis.^58^

Remarkably, TP53BP1, a marker for DNA damage repair, was highly expressed in nonresponders. DNA repair proteins like TP53BP1 serve as markers for DNA double-strand breaks (DSBs), which are crucial for efficient radiotherapy as they lead to cell death.^59^ Yang et al. showed the important role of TP53BP1 in a radiosensitivity model system using isogenic HCT116 and SW480 colorectal cancer cell lines bearing wild-type or various mutant KRAS isoforms.^60^ KRAS mutations may result in increased DNA damage, and upregulation of TP53BP1 was found to be associated with increased nonhomologous end-joining (NHEJ). Thereby, the authors suggested that targeting TP53BP1 or NHEJ may represent novel strategies to selectively abrogate KRAS mutation-mediated radioresistance.^60^

COTL1, a cytoskeleton-associated protein, and DCPS also belong to the six specifically regulated proteins. While COTL1 has already been proposed as a potential prognostic biomarker in glioma^61^ and breast cancer,^62^ Dimberg et al. showed that DCPS mRNA and protein expression were significantly up-regulated in patients with colorectal cancer.^63^

The specific up-regulation of TOM1, CAPNS1, TP53BP1, COTL1, HS1BP3, and DCPS in nonresponders compared to complete responders to radiation therapy is striking in the background of their important biological roles in autophagic activity, DNA damage repair, and radio-/chemotherapy resistance mechanisms. They represent promising candidate markers for the prognosis of radiation response in colorectal cancer.

The retrospective analysis of FFPE tumor and control tissue samples from colorectal cancer patients further offered the possibility of investigating the impact of the surrounding stroma on radioresistance mechanisms. Therefore, comparisons of tumor and control tissue of patients were performed separately for nonresponders and responders (Figure 4). Thus, a remarkable mechanism on how the tumor-surrounding tissue might impact the sensitivity of tumors to radiotherapy became apparent. This was indicated by the up-regulation of CD55 in the control tissue of nonresponders, a protein that is known to be highly expressed in the tumor environment, playing a specific role in the suppression of adaptive immune responses.^35,36^ The concomitant up-regulation of complement-associated proteins like complement component C8 alpha and beta chain (C8A and C8B) in the tumor tissue of responders but not in the tumor tissue of nonresponders may indeed result from higher levels of CD55 in the stroma of nonresponders, thereby inhibiting the central driver of the complement cascade, C3 convertase.^37^ High expression of CD55­(+) in tumor cell populations has already been shown to contribute to tumorigenicity and radioresistance in various cancer types like cervical cancer, breast cancer, and neuroblastoma.^64–66^ Only recently, the potential of complement regulators such as CD55 as therapeutic targets has been discussed due to their important role in complement activation and the tumor microenvironment.^67^

## Conclusions

This study clearly demonstrates the great potential of using FFPE tissue samples of CRC patients for proteome profiling in order to investigate pathophysiological mechanisms in relation to radioresistance. The strategy of analyzing not only tumor tissue alone but also in combination with corresponding control tissue from the same patient allowed us to identify specific marker molecules potentially predicting response to radiation therapy in colorectal cancer patients based on molecular mechanisms taking place in tumor as well as tumor-surrounding tissue. More precisely, TOM1, CAPNS1, TP53BP1, COTL1, HS1BP3, and DCPS are promising candidates as potential prognostic markers for radiation response in CRC due to their functional role in autophagy as well as DNA damage repair.

## Limitations of the Study

In this study, FFPE tissue samples were used to investigate proteins potentially related to the pathophysiological mechanisms underlying resistance to chemoradiation in colorectal cancer patients. During tissue fixation with formaldehyde, proteins are chemically cross-linked in comparison to fresh frozen tissue samples. This typically results in lower numbers of protein identifications. Further, due to high interindividual differences in the patient cohort, the numbers of significantly regulated proteins were rather low. Nevertheless, those significantly regulated proteins may thus reflect the most robust differences between the study groups, making them strong candidates as potential prognostic markers for radiation response in CRC. Since the present study was solely retrospective, the predictive power of potential marker molecules, as well as their suitability as therapeutic targets to improve response rates to neoadjuvant chemoradiation, has to be validated in a larger and independent prospective study.

## Supplementary Material





## Data Availability

All proteomics data were submitted to the ProteomeXchange Consortium (http://proteomecentral.proteomexchange.org) and are available in the PRIDE partner repository^68^ with the data set identifier PXD060201.

## Notes

Protein list for paired *t* test statistics of tumor and control FFPE tissue samples; uniprot accession, gene names as well as adj. *p*-values and log2 fold-changes from the comparison of tumor and control tissue is listed separately for each protein; number of identified peptides, unique peptides, sequence coverage, and log2-transformed label-free quantification (LFQ) values are listed for each sample (Table S1) (XLSX)
Protein list for unpaired *t* test statistics between nonresponders (NR) and responders (R) to radiation therapy as well as from nonresponders to the subtypes of responders, i.e., partial, nearly complete and complete responders; uniprot accession, gene names as well as adj. *p*-values and log2 fold-changes for each comparison relisted separately for each protein; number of identified peptides, unique peptides, sequence coverage, and log2-transformed label-free quantification (LFQ) values are listed for each sample (Table S2) (XLSX)

## Abbreviations

CAN: acetonitrile
AJCC/CAP: American Joint Committee on Cancer and College of American Pathologists
BMI: body mass index
cCR: clinical complete response
CEACAM: carcinoembryonic antigen-related cell adhesion molecules
CRC: colorectal cancer
CRT: chemoradiotherapy
DTT: dithiothreitol
FA: formic acid
FDR: false discovery rate
FFPE: formalin-fixed paraffin-embedded
IAA: iodoacetamide
LC-MS: liquid chromatography–mass spectrometry
LFQ: label-free quantification
MCM: minichromosome maintenance protein complex
NAT: neoadjuvant therapy
nCRT: neoadjuvant chemoradiotherapy
NR: nonresponders
OS: overall survival
pCR: pathological complete response
R: responders
RT: radiotherapy
SDS: sodium dodecyl sulfate
TEAB: triethylammonium bicarbonate
TNT: total neoadjuvant therapy
UICC: Union Internationale Contre le Cancer
